# 环状RNA CircHIPK3在非小细胞肺癌中的研究进展

**DOI:** 10.3779/j.issn.1009-3419.2024.106.20

**Published:** 2024-08-20

**Authors:** Yuheng WANG, Liang CHEN

**Affiliations:** 210000 南京，南京医科大学第一附属医院胸外科; Department of Thoracic Surgery, First Affiliated Hospital of Nanjing Medical University, Nanjing 210000, China

**Keywords:** 环状RNA, HIPK3, 肺肿瘤, 竞争性内源性RNA, 生物标志物, Circular RNA, HIPK3, Lung neoplasms, Competitive endogenous RNA, Biomarkers

## Abstract

肺癌是全世界发病率和致死率最高的恶性肿瘤之一，虽然针对肺癌的靶向、免疫治疗等新兴治疗手段取得了显著进展，但如何治愈肺癌仍是一项备受瞩目的话题。其中环状RNA（circular RNA, circRNA）作为最近发现的一类具有共价闭合结构的环状RNA，具有结构稳定、序列保守及疾病特异性表达等特点。当前医学前沿揭示了circRNA的失调与多种癌症的发展过程有关。其中，研究热点之一的环状RNA HIPK3（circular RNA HIPK3, circHIPK3）是一种致癌基因，主要起源于基因HIPK3的第二个外显子。越来越多的证据表明circHIPK3参与了非小细胞肺癌（non-small cell lung cancer, NSCLC）等多种恶性肿瘤的发展，并且circHIPK3的异常表达与NSCLC的疾病机制、诊断、治疗以及预后密切相关。本文旨在对circHIPK3在NSCLC中的最新研究进展进行综述，以推动肺癌的精准诊疗。

## Abstract (en)

Lung cancer ranks among the most prevalent and deadliest malignancies worldwide. Despite significant strides in targeted therapies and immunotherapy for lung cancer, curing the disease remains a highly prioritized issue. Circular RNAs (circRNAs), recently discovered RNA molecules characterized by covalently closed loop structures, possess features such as structural stability, sequence conservation, and disease-specific expression. Cutting-edge medical research has linked circRNA dysregulation to the progression of various cancers. Among these, circular RNA HIPK3 (circHIPK3), an oncogenic gene primarily derived from the second exon of the HIPK3 gene, has emerged as a focal point of investigation. Increasing evidences suggest that circHIPK3 is involved in the development of non-small cell lung cancer (NSCLC) and other malignancies. Aberrant expression of circHIPK3 is closely associated with the disease mechanisms, diagnosis, treatment, and prognosis of NSCLC. This review discusses the latest research advancements on circHIPK3 in NSCLC, aiming to promote precise diagnosis and treatment of lung cancer.

肺癌是一种主要起源于支气管黏膜上皮的恶性肿瘤^[1]^，在我国癌症发病率中占据很高的比例，也是目前肿瘤导致死亡的主要原因^[2]^。在肺癌的病理学分类中，可分为小细胞肺癌（small cell lung cancer, SCLC）和非小细胞肺癌（non-small cell lung cancer, NSCLC）两种主要的细胞学类型^[2]^。其中NSCLC更为常见，约占肺癌患者的85%^[3]^。目前，随着外科手术、化疗、放疗、免疫治疗等治疗方法的综合应用，早期肺癌的治愈率得到了显著的提升^[4]^，但晚期NSCLC患者的预后仍然较差，其5年生存率低于20%^[5]^。许多患者在确诊时已处于晚期，常常因为发生转移而错过手术时机^[6]^。因此，当务之急是进一步探索NSCLC发生及进展的分子机制，从而提高早期肺癌的生存率。

20世纪70年代，研究人员第一次在植物RNA病毒中检测出环状RNA（circular RNA, circRNA），起初它被认为是转录本错误剪接而产生的副产品^[7]^。然而，随着转录组测序技术的进展，科学家们发现circRNA是由RNA聚合酶II转录形成的内源性RNA，可分为非编码和编码circRNA，相较于线性RNA，circRNA的闭合环状结构使其对核酸外切酶具有更强的抵抗性^[8]^。由于circRNA有着结构稳定、疾病特异性表达和抗降解性等特点，很快掀起了对非编码RNA的研究浪潮^[9]^。其中circRNA HIPK3（circ RNA HIPK3, circHIPK3）作为一种新发现的circRNA，与NSCLC的诊断、治疗和预后评估密切相关^[10]^。本篇综述通过总结国内外文献，就circHIPK3在NSCLC中的作用机制及研究进展进行总结，为NSCLC的早期诊断和治疗提供新的方法。

## 1 CircHIPK3概述

CircHIPK3起源于同源结构域相互作用蛋白激酶3（homeodomain-interacting protein kinase 3, HIPK 3）基因的第二个外显子（图1），与大多数circRNA一样，circHIPK3主要定位于细胞质中，且其在肺、乳腺、胰腺等其他器官的组织细胞质中高度表达^[11]^。既往研究^[11]^证实了circHIPK3可以通过以下分子机制参与肿瘤的发生发展：（1）与蛋白质相互作用：circHIPK3可以与某些蛋白质如RNA结合蛋白（RNA-binding protein, RBP）相互作用，干扰或增强其功能；（2）基因转录调节：在转录过程中发挥作用，与聚合酶II复合物相互作用，下调特定基因的表达；（3）调节表观遗传；（4）运输物质信息。除此而外，circHIPK3参与了多种癌症的发生和发展的过程，在肺癌^[12⇓⇓⇓-16]^、膀胱癌^[17]^、胃癌^[18,19]^、肾癌^[20]^、前列腺癌^[21,22]^、口腔鳞状细胞癌^[23]^、卵巢癌^[24]^、宫颈癌^[25]^、结直肠癌^[26]^、肝细胞癌^[27]^、胰腺癌^[28]^、心脏纤维化^[29]^、甲状腺癌^[30]^等疾病的发生发展中起到了重要的作用（表1^[12⇓⇓⇓⇓⇓⇓⇓⇓⇓⇓⇓-24,26]^）。

**图1:**
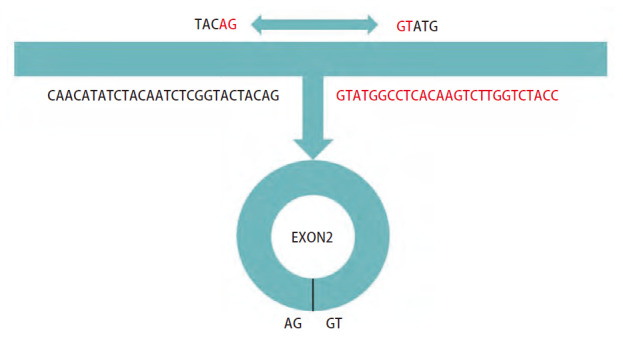
CircHIPK3的反向剪接位点序列

**表1:** CircHIPK3在多种肿瘤中的表达、机制以及功能

Cancer types	Expression	Function	Related genes	Ref
Lung cancer	Up	Proliferation, migration, invasion, apoptosis, autophagy	STAT3, CDK4, SphK1, IGF-1, FOXM1, PRKAA, AMPKα, BDNF	^[12⇓⇓⇓-16]^
Bladder cancer	Down	Migration, invasion, angiogenesis, metastasis	HPSE, VEGF	^[17]^
Gastric cancer	Up Down	Proliferation Unknown	BDNF, TCF4 CDK6, WNT1	^[18,19]^
Renal cancer	Up	Migration, invasion	CXCL13, vimentin	^[20]^
Prostate cancer	Up	Invasion, angiogenesis	MCL1	^[21,22]^
Oral squamous cell carcinoma	Up	Proliferation	Unknown	^[23]^
Epithelial ovarian cancer	Up	Unknown	Unknown	^[24]^
Colorectal cancer	Up	Proliferation, migration, invasion	FMNL2	^[26]^

STAT3: signal transducer and activator of transcription 3; CDK4: cyclin-dependent kinases 4; SphK1: sphingosine kinase 1; IGF-1: insulin like growth factor-1; FOXM1: forkhead box protein M1; PRKAA: people 5＇AMP activated protein kinase α2 catalytic subunit; AMPKα: adenosine activated protein kinase α; BDNF: brain derived neurotrophic factor; HPSE: recombinant heparanase; VEGF: vascular endothelial growth factor; TCF4: transcription factor 4; CDK6: cyclin-dependent kinase 6; CXCL13: C-X-C motif chemokine 13; MCL1: myeloid cell leukemia 1; FMNL2: formin-like protein 2.

## 2 CircHIPK3与NSCLC的关系

### 2.1 CircHIPK3作为NSCLC的新型诊断生物标志物

由于NSCLC的早期诊断检查费用昂贵，且临床影像学诊断的准确性有限，至今不能取得满意的效果，因此寻找适合的NSCLC的诊断生物标志物具有重要意义^[31]^。既往研究^[32,33]^表明circRNA可作为早期诊断的新兴标志物。细胞外囊泡（extracellular vesicles, EVs）是指含有各种核酸如微小RNA（microRNA, miRNA）和circRNA的微小囊泡，在基因表达中充当调节剂并且可作为疾病诊断的生物标志物^[34,35]^。为了研究EVs来源的circHIPK3在肺癌诊断中的作用，Zhu等^[32]^通过对52例NSCLC患者进行实时荧光定量聚合酶链式反应（real-time quantitative polymerase chain reaction with fluorescent detection, qPCR）来检测circHIPK3的表达水平。结果显示，与健康对照相比circHIPK3在NSCLC中表达显著上调（P<0.0001），并且circHIPK3的水平与肿瘤原发灶-淋巴结-转移（tumor-node-metastasis, TNM）分期呈正相关（P<0.0001）。受试者工作特征（receiver operating characteristic, ROC）分析中曲线下面积（area under the curve, AUC）是评价circRNA诊断价值的主要指标^[36]^。Zhu等^[32]^发现circHIPK3在区分NSCLC患者和健康人群方面具有高诊断效率，其AUC为0.897。综上所述，这些结果表明EVs来源的circHIPK3适合作为NSCLC早期诊断的生物标志物。然而本项研究选取的健康对照样本量较少，未来需要扩大样本量以进行下一步探索。

### 2.2 CircHIPK3在NSCLC中的预后价值

晚期肺癌由于死亡率高和预后差成为临床诊治工作的难点之一^[37]^。为了探究circHIPK3在NSCLC中的预后价值，Yu等^[38]^发现在NSCLC组织中circHIPK3表达丰富，能促进肺癌细胞的迁移和增殖，通过Kaplan-Meier生存曲线分析和多因素比例风险回归模型研究发现circHIPK3可作为NSCLC总生存期的独立预后因素。Chen等^[14]^研究了54例NSCLC患者的组织样本，使用qPCR检测发现肺癌组织中的circHIPK3高表达且circHIPK3与NSCLC患者预后之间有相关性。后续的研究^[14]^表明了circHIPK3和线性RNA HIPK 3（linHIPK3）之间的比值（C/L值）在一定程度上反映了肿瘤的自噬水平，尤其在晚期患者中，高C/L值（>0.49）是预后不良的指标。同年，Lu等^[16]^通过qPCR检测30例NSCLC患者的肿瘤样本，发现其与正常组织相比circHIPK3在NSCLC组织中显著上调（P<0.005）。Gu等^[12]^的团队评估了circHIPK3在肺癌中的预后意义，他们根据NSCLC组织中circHIPK3表达的中值，将45例患者分为circHIPK3低表达组（n=22）和circHIPK3高表达组（n=23），进一步分析发现circHIPK3的表达与肿瘤大小（P=0.0003）、TNM分期（P=0.0003）和淋巴结转移（P=0.0009）有紧密联系，是与肺癌的预后高度相关的因素之一。

总而言之，以上研究进一步验证了circHIPK3作为致癌基因并且具有作为NSCLC预后因子的临床价值，为患者生存预后分析提供了新的思路。值得注意的是，未来需要更加灵敏和准确的检测方法将circHIPK3投入临床应用中。由于样本量的限制导致了结论的普适性不足，因此仍需要更大规模的研究来验证circHIPK3的预后价值。

### 2.3 CircHIPK3在NSCLC中的生物学作用

为了探究circHIPK3在NSCLC恶性生物学行为中的作用，Hong团队^[15]^通过细胞增殖成像分析实验（5-ethynyl-2'-deoxyuridine, EdU）及Transwell侵袭测定实验等检测circHIPK3在NSCLC细胞侵袭和增殖中的功能，结果显示下调circHIPK3的表达显著抑制了NSCLC细胞的增殖和侵袭；该团队进一步研究发现circHIPK3可作为miR-107海绵，促进脑源性神经营养因子（brain-derived neurotrophic factor, BDNF）细胞增殖，而BDNF在促进肿瘤的转移和增殖能力中起着重要作用^[39]^，明确了circHIPK3在NSCLC中的促癌作用。Katopodi等^[40]^从免疫学的角度发掘了CD163^+ ^M2巨噬细胞和circHIPK3/PTK2轴之间相互作用的分子机制，揭示了其促进肺癌的免疫抑制性的生物学功能。具体而言，外泌体中cicHIPK3/PTK2表达的上调促进了Kras驱动的肿瘤内异质性，并加速了淋巴结转移。Lu团队^[16]^的实验表明circHIPK3加速了血管生成，促进了NSCLC的恶性生物学行为，靶向作用于叉头框蛋白M1（forkhead box protein M1, FOXM1）促进NSCLC的上皮间充质转化（epithelial-mesenchymal transition, EMT），加速了肿瘤的转移及侵袭。同年，Gu等^[12]^发现敲降circHIPK3可以减弱NSCLC细胞的葡萄糖消耗，降低乳酸盐和己糖激酶2（hexokinase 2, HK2）的活性，而HK2是糖酵解过程中的必需酶^[41]^，由此说明circHIPK3的沉默抑制了糖酵解，同时促进了肺癌细胞的凋亡。综上所述，circHIPK3参与NSCLC中的多种恶性生物学行为，加速了NSCLC细胞的增殖和侵袭等，并调控了糖酵解过程。此外，circHIPK3还参与了免疫失调和免疫抑制，产生了免疫抑制性肿瘤促进网络^[14]^；促进了血管形成等不良生物学行为。这些研究结论阐明了circHIPK3在NSCLC生物学行为发展中的重要作用，使我们对NSCLC的致癌机制有了新的认识，并为治疗提供了新思路。

## 3 CircHIPK3的促癌机制

### 3.1 竞争性内源RNA（competing endogenouse RNA, ceRNA）机制

ceRNA是由Pier Paolo Pandolfi团队于2011年首次提出的概念^[42,43]^，其对基因之间的调控有着指导性的意义。研究^[44,45]^发现各种类型的RNA，包括mRNA、circRNA、lncRNA、miRNA等之间可以互相竞争调节。ceRNA即通过这种竞争性结合关系从而调节下游基因的表达。通过对竞争性调控网络的广泛研究，对环状非编码RNA有了更多的认识，加速了对肺癌发生机制的探索。

研究^[46]^证实了circRNA可以结合miRNA以抑制其作为竞争性内源RNA或miRNA海绵的功能，考虑circHIPK3可能作为ceRNA来调控基因表达^[46]^（图2），如circHIPK3可以与miRNA反应元件（microRNA response element, MRE）结合，从而起负性调节作用。早在2017年，Tian等^[13]^的研究表明circHIPK3通过结合miR-379，上调胰岛素样生长因子1（insulin-like growth factor 1, IGF-1）的表达。IGF-1是一种重要的细胞有丝分裂促进剂，调控细胞生长与分化^[47]^。circHIPK3通过circHIPK3/miR-379/IGF-1轴促进肺癌细胞恶性生物学进展。随后Yu等^[38]^发现circHIPK3可以作为miR-124海绵，调节NSCLC细胞下游靶标如鞘氨醇激酶1（sphingosine kinase 1, Sphk1）、周期蛋白依赖激酶4（cyclin-dependent kinase 4, CDK4）和信号转导因子和转录激活因子3（signal transducer and activator of transcription 3, STAT3）的水平，从而有效促进肺癌细胞的增殖和侵袭能力。另一项研究^[16]^发现circHIPK3调节miR-149，而miR-149通过结合到3′非翻译区（untranslated region, UTR）与FOXM1相互作用。FOXM1是 Forkhead box家族的转录因子之一，在实体肿瘤中调控细胞周期、增殖和衰老等多个生物学过程^[48]^，研究结果表明circHIPK3通过circHIPK3/miR-149/FOXM1调节轴促进NSCLC的恶性生物学进展。Chen等^[14]^证明了circHIPK3在肺癌细胞中大量表达并发挥致癌作用。该研究团队发现，缺乏circHIPK3则抑制了肿瘤自噬的生物行为，即沉默circHIPK3通过miR-124-3p/STAT3/PRKAA/AMPKα信号通路显著诱导肿瘤自噬，为肺癌的免疫治疗提供新的视角。其他研究^[15]^发现了circHIPK3可作为miR-107的海绵发挥作用，并促进了miR-107直接靶向的BDNF表达，提高肿瘤转移和增殖能力。Gu等^[12]^的研究揭示了circHIPK3作为miR-381-3p的分子海绵，调节AKT/mTOR通路。而AKT/mTOR通路已经被证实与细胞生长、代谢和存活密切相关，可促进肺癌的恶性进展^[49]^。

**图2:**
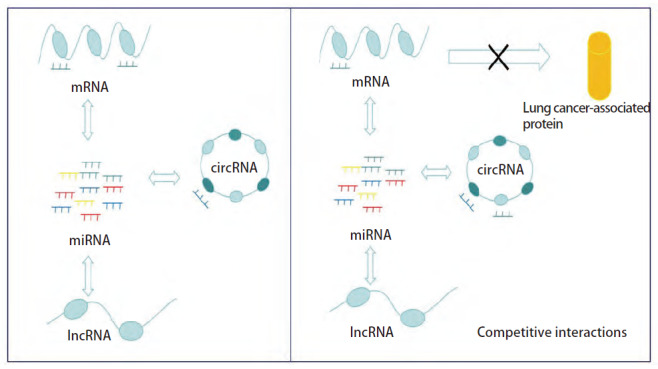
CircRNA作为ceRNA促进NSCLC进展

近年来，越来越多的研究^[12⇓⇓⇓⇓⇓⇓⇓⇓⇓⇓-23,25⇓⇓⇓⇓-30]^报道了circHIPK3通过ceRNA调控网络促进NSCLC进展，然而肿瘤的发生机制是极其复杂的，现有的研究集中于circRNA的miRNA海绵功能上，研究者如果仅仅局限在零散的miRNA到靶基因的调节通路上很难对癌症的发展有全面的认知，因此需要引入更多因子来丰富ceRNA的复杂调控网络^[50]^，使其更加立体化，从而真正提高早期肺癌的诊治水平。

### 3.2 其他促癌机制研究

除了广泛研究的miRNA海绵功能，circHIPK3还可以通过多种免疫学机制调控肺癌的生物学进展^[51]^。浸润肿瘤组织或聚集在实体肿瘤微环境中的巨噬细胞被定义为肿瘤相关巨噬细胞（tumor-associated macrophages, TAMs）^[52]^，TAMs调节了肿瘤生长、血管浸润、免疫应答过程。2021年Katopodi等^[40]^在研究中发现，circHIPK3在Kras介导的信号传导通路中促进了肿瘤微环境中TAMs的浸润，引发免疫环境改变，导致NSCLC进展^[53]^。研究者通过qPCR在NSCLC患者样品和细胞系中确定了circHIPK3的表达模式，发现其主要与M2巨噬细胞相关^[54]^，并且定位于肿瘤巢和肿瘤的浸润周边。接着通过细胞凋亡染色和流式细胞术测定M2巨噬细胞极化和髓系抑制性细胞（myeloid-derived suppressor cells, MDSC）亚群分析（Gr1^−^/CD11b^−^, Gr1^−^/CD11b^+^）发现MDSC亚群通过circHIPK3/PTK2通路触发了M2依赖性免疫应答，继而形成了免疫抑制性肿瘤促进网络。总之，circHIPK3调节肺癌免疫抑制性网络传导，并且通过circHIPK3/PTK2通路促进了NSCLC转移进展，为NSCLC的治疗靶点提供了方向。另一项免疫学机制研究^[14]^发现降低circHIPK3的表达会诱导肺癌细胞自噬，即抑制了NSCLC细胞自我降解和循环利用胞内组分的过程。鉴于HIPK3蛋白作为自噬调节剂的作用已经得到证实^[55]^，Chen等^[14]^考虑circHIPK3是否通过相同的机制影响NSCLC细胞，研究者们在circHIPK3敲低后观察到自噬微管相关蛋白轻链β3（microtubule-associated protein 1 light chain 3 beta, LC3B）表达增加、LC3B-I/II加速转化降解，而LC 3B是一种泛素样蛋白，存在非脂化形式LC3B-I和脂化形式LC3B-II，可以作为自噬诱导的敏感标志物^[56]^。研究结果表明circHIPK3调控了肺癌细胞的自噬过程，患者生存数据也证实了维持高自噬流改善了NSCLC患者的预后。综上所述circHIPK3通过免疫逃逸以及自噬调节促进NSCLC进展，为肺癌的免疫治疗提供了新的思路。

## 4 CircHIPK3与靶向药物抗性的机制研究

随着对NSCLC的认识不断深入，治疗手段日益丰富，形成了以手术、放疗、化疗、靶向治疗和生物免疫疗法为主的综合治疗方案^[57]^。目前美国国立综合癌症网络（National Comprehensive Cancer Network, NCCN）指南认为手术治疗是肺癌的首选方案^[58]^。然而对于一些无法手术或术后复发的患者，分子靶向治疗的发展提高了患者的远期生存率。

表皮生长因子受体（epidermal growth factor receptor, EGFR）是一种酪氨酸激酶受体，在多种肿瘤中异常表达，可促进肿瘤进展^[59]^。吉非替尼是一种EGFR受体的抑制剂^[60]^，最早应用于临床一线抗肿瘤治疗，既往的研究^[61]^表明吉非替尼适用于局部晚期或转移性NSCLC患者的治疗，可以有效改善患者的预后，但在使用前必须检测EGFR基因。吉非替尼通过以下三条路径发挥作用^[62]^：（1）竞争性结合于EGFR-络氨酸激酶（tyrosine kinases, TK）催化区域上的Mg-ATP位点，阻断下游传导；（2）抑制有丝分裂原活化蛋白激酶活化，促进细胞凋亡；（3）抑制血管生成。通过以上机制，吉非替尼特异性阻碍NSCLC的进展过程，抑制肺癌细胞的传导、浸润以及定位，从而杀伤癌细胞。另外，有报道^[63]^称吉非替尼联合化疗可显著提高肺癌患者的治疗效果。然而在实际临床应用中，吉非替尼耐药仍然是肺癌分子靶向治疗中的一个主要难题，明显降低了治疗效果。涉及吉非替尼耐药的机理在很大程度上尚不清楚，目前研究^[64]^主要集中在吉非替尼耐药性的生物学机制上。

Song的团队^[65]^为了研究circHIPK3调控肺癌细胞耐药性的机制，检测了110例肺癌患者的肿瘤组织和癌旁组织中circHIPK3的表达，他们构建了对吉非替尼产生耐药的NSCLC细胞系，检测在不同状态下细胞凋亡情况。研究结果表明，肿瘤直径≥3 cm的患者中circHIPK3的相对表达显著低于肿瘤直径<3 cm的患者（P<0.05）；TNM分期在II到III期患者circHIPK3的相对表达明显低于TNM分期I期患者（P<0.05）；有淋巴结转移组的circHIPK3表达显著低于无淋巴结转移患者（P<0.05）。在不同TNM分期患者的肺癌组织中，只有6例患者高表达，其余104例患者低表达。总之，circHIPK3在耐药患者组织中存在显著的低表达，其低表达促进NSCLC细胞对吉非替尼的耐药性。通过流式细胞术凋亡检测发现，吉非替尼介导的耐药肺癌细胞株凋亡率显著降低，这与Zhou的团队^[66]^的研究结果相似。Song的团队^[65]^继续探究发现circHIPK3可以通过靶向miR-124调控多药耐药相关蛋白4（multidrug resistance protein 4, MRP4）的表达，促进NSCLC细胞对吉非替尼的耐药性以及肺癌的恶性生物学行为。此外Song等^[65]^将circHIPK3和mRNA共表达分析，通过结合转录组数据来预测它们之间的相互作用关系，检测到白细胞介素1（interleukin-1, IL-1）和肿瘤坏死因子（tumor necrosis factor, TNF）的表达同时增加，调控了肺癌细胞对吉非替尼的耐药性。然而，在这些研究中，由于部分患者临床基线数据不完整，可能会在一定程度上导致样本偏倚；同时，这项研究选取的样本量较小，代表性不够全面。因此，还需要进一步扩大样本量。最终如何突破NSCLC的耐药性，还需要融合肿瘤微环境、EGFR的突变与内化等^[67]^更多的机制，但这项研究为NSCLC的靶向治疗带来了新的希望。

## 5 总结与展望

随着RNA测序技术的进步和基因信息学的快速发展，circRNA在检测疾病状态和基因治疗方面的潜在用途正在不断被挖掘^[68]^。鉴于大量不同的circRNA及其潜在的组织特异性功能，有必要深入了解它们参与和调节的复杂基因网络。其中，circHIPK3作为新兴的研究热点，在NSCLC诊断、预后和治疗评估方面显露出重要价值^[69]^。本文综述了circHIPK3作为一种致癌基因，与NSCLC的早期诊断、生物学功能（如细胞周期、增殖、侵袭、自噬）、预后（如肿瘤分期、淋巴结转移）和药物耐药性等密切相关（表1^[12⇓⇓⇓⇓⇓⇓⇓⇓⇓⇓⇓-24,26]^），参与了蛋白质、基因的表达调控等病理生理过程。在机制方面，circHIPK3通过直接结合miRNA调控基因表达、作为ceRNA激活各种信号通路、与转录因子的结合、调节肿瘤微环境、促进肿瘤免疫逃逸等途径促进NSCLC进展，显著影响患者的预后。

与其他非编码RNA研究相比，NSCLC中circRNA的研究仍处于早期阶段。由于技术限制，目前研究集中在circHIPK3作为miRNA海绵的作用上，对circHIPK3的蛋白编码功能等研究甚少。未来需要进一步的研究来探索circHIPK3在NSCLC中的相互作用网络，包括miRNA、mRNA和蛋白质降解途径的参与等。其次，关于NSCLC的液体活检研究甚少，以后有望将circHIPK3与不同类型标志物相结合，为NSCLC的早期诊断、治疗的选择以及病程的预后提供新的思路。鉴于circHIPK3独特的稳定性，人工circHIPK3构建和干扰技术有助于突破抗肿瘤药物的耐药性，促进药物载体和潜在治疗靶点的进展。随着基因测序技术的优化和广泛运用、生物信息学新方法的建立，将更加系统全面地理解circHIPK3潜在的作用机制，加速其在NSCLC诊断和治疗中的临床转化。未来新型靶向材料的问世^[70]^以及免疫学的突破将促进对circHIPK3产生新的认识，从而推动精准医学的进步。
